# A case report of type VI dual left anterior descending coronary artery anomaly presenting with non-ST-segment elevation myocardial infarction

**DOI:** 10.1186/1471-2261-12-101

**Published:** 2012-11-13

**Authors:** Yonggu Lee, Young-Hyo Lim, Jinho Shin, Kyung-Soo Kim

**Affiliations:** 1 Department of Cardiology, Hanyang University Hospital, Wangsipri Street 222, Seongdong-Gu, Seoul, South Korea

**Keywords:** Type VI dual LAD anomaly, Percutaneous coronary intervention, Computed tomographic coronary angiography

## Abstract

**Background:**

Type VI dual left anterior descending artery (LAD) is a rare coronary anomaly, the first case of which has recently been described. This is the first report of type VI dual LAD anomaly in which the patient presented with non-ST-segment elevation myocardial infarction and percutaneous coronary intervention was performed in the anomalously originating LAD.

**Case presentation:**

A 52-year-old man with diabetes, hypertension and hyperlipidemia presented with chest pain without ST elevation on EKG, although the patient’s troponin I level was elevated. Coronary angiography revealed a short LAD originating from the left main coronary artery and a long LAD originating from the proximal portion of the right coronary artery (RCA). Three-dimensional reconstruction of computed tomography of images revealed that the long LAD originated from the proximal RCA and coursed between the right ventricular outflow tract (RVOT) and the aortic root before entering the mid anterior interventricular groove. The high take-off RCA originated underneath the RVOT, pointing downwards and forming an acute angle with the proximal portion of the long LAD. The anomalous long LAD displayed significant stenosis. We performed successful percutaneous coronary intervention (PCI) in the anomalous artery.

**Conclusion:**

With accurate understanding of the coronary anatomy and appropriate hardware selection, successful PCI can be performed in the in the long LAD in patients with type VI dual LAD anomaly.

## Background

Accurate assessment of the coronary anatomy is crucial for successful percutaneous coronary interventions (PCI) in anomalously originating coronary arteries. Type VI dual left anterior descending artery (LAD), recently reported by Maroney et al. [1], is an anomaly in which a short LAD originates from the left main coronary artery (LMCA) and a long LAD originates from the right coronary artery (RCA). The long LAD passes between the right ventricular outflow tract (RVOT) and the aortic root. We report a case of type VI dual LAD anomaly presenting with non-ST-segment elevation myocardial infarction in which PCI on the anomalous long LAD was successfully performed.

## Case presentation

A 52-year old man with hypertension, type 2 diabetes and dyslipidemia presented with sustained chest pain. Electrocardiography showed flattened T waves in lateral and inferior leads and the patient’s serum troponin I was increased. Echocardiography showed inferior wall hypokinesia with preserved left ventricular ejection fraction. The patient was diagnosed with non-ST-segment elevation myocardial infarction and was admitted to the coronary care unit.

**Figure 1:**
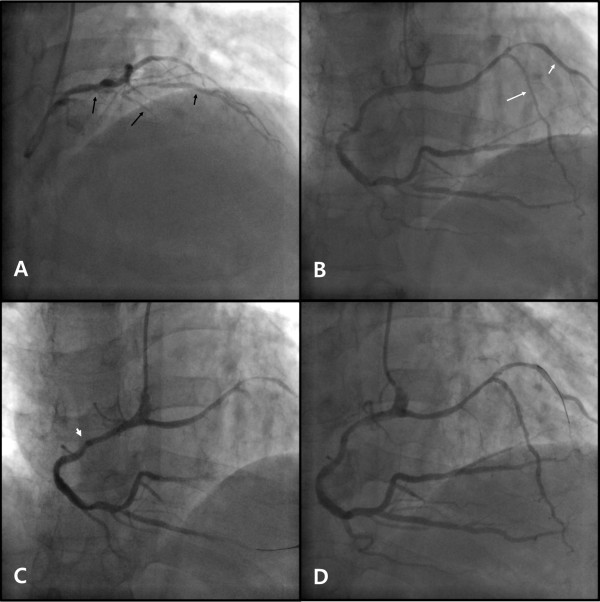
**Coronary angiography and percutaneous coronary intervention.A**) Short LAD (long black arrow) originating from the LMCA, giving rise to the D1 (short black arrow). There is significant stenosis in the proximal portion of the short LAD and the proximal portion of the D1. (**B**) Long LAD (long white arrow) originating from the proximal RCA, coursing left, then turning downwards to the apex, giving rise to the D2 (long white arrow). There is significant stenosis of the distal RCA, the proximal PDA, the mid portion of the long LAD and the proximal portion of the D2. (**C**) Deep engagement (arrowhead) with a 6 Fr MP guiding catheter to deliver stents to the distal RCA. (**D**) Balloon angioplasty with a 6 Fr JR 4.0 guiding catheter at the mid portion of the long LAD and the proximal portion of the D2. LAD, left anterior descending artery; LMCA, left main coronary artery; D1, first diagonal artery; RCA, right coronary artery; PDA, posterior descending artery; D2, second diagonal artery; 6 Fr, 6 French; MP, Multipurpose; JR, Judkins right.

On the second day after admission, coronary angiography was performed. Left coronary angiography revealed a short LAD originating from the LMCA, giving rise to proximal septal branches and the first diagonal artery (D1) (Figure 1A). There was significant stenosis of the proximal portions of the short LAD and the D1, and there appeared to be total occlusion of the mid portion of the short LAD. The left circumflex artery originated normally from the LMCA and showed no significant stenosis. Right coronary angiography revealed a dominant RCA with a long LAD originating from its proximal portion (Figure 1B). The RCA originated above the right coronary sinus and pointed downwards. The long LAD coursed left until turning downwards to the apex, giving rise to the second diagonal artery (D2) at the turning point. No bridging phenomenon was observed in systole in the traversing portion of the long LAD. There was significant stenosis of the mid portion of the long LAD, the proximal portion of the D2, the distal RCA and the proximal portion of the posterior descending artery.

PCI was performed using a right trans-radial approach with a 6-French sheath. Deep engagement with a Multipurpose (MP) guiding catheter (Cordis Corporation, Bridgewater, NJ, USA) was required to deliver a balloon and stents through the downward-oriented RCA ostium (Figure 1C). Xience Prime everolimus-eluting stents (Abbott Laboratories, Illinois, USA) were implanted in the distal RCA and the posterior descending artery (stent size 3.0 × 24 mm and 3.5 × 24 mm, respectively). Successful balloon angioplasty was performed with a Judkins right (JR) 4.0 guiding catheter (Cordis Corporation, Bridgewater, NJ, USA) and a 2.5 × 20 mm Ikazuchi balloon (Kaneka Medical Products, Nagoya, Japan) in the long LAD and the D2 (Figure 1D). The procedure was terminated at this point because of concern about the difficulty of delivering a stent through the acute angle between the proximal RCA and the traversing portion of the long LAD with poor guiding catheter support. Finally, a Judkins left 4.0 guiding catheter (Cordis Corporation, Bridgewater, NJ, USA) was used in the short LAD and a 3.0 × 28 mm Xience Prime stent was implanted in the short LAD, crossing over the D1.

**Figure 2:**
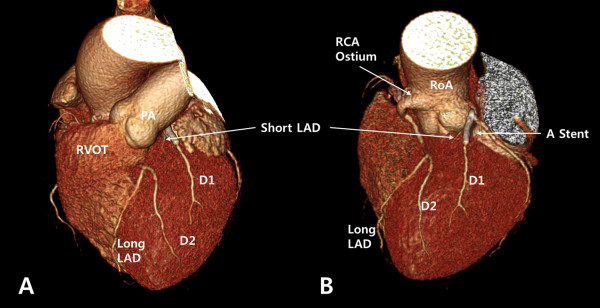
**Computed tomographic coronary angiography.** (**A**) LAO cranial view showing the dual LAD anomaly with the short LAD on the proximal AIVG, giving rise to the D1 and the long LAD entering the mid AIVG giving rise to the D2. (**B**) LAO cranial view with the RVOT removed, showing the short LAD originating from the left main coronary artery and the long LAD originating from the proximal RCA. The proximal RCA is taking off above the RCS and the long LAD is branching from the proximal RCA, passing between the RVOT and the aortic root. A stent can be observed from the proximal portion of the short LAD to the D1. The D2 is the dominant artery in the anterior wall of the left ventricle. LAO, left anterior oblique; LAD, left anterior descending artery; LMCA, left main coronary artery; RCA, right coronary artery; AIVG, anterior interventricular goove; D1, first diagonal artery; D2, second diagonal artery; RVOT, right ventricular outflow tract; PA, pulmonary artery; RoA, root of aorta.

On the third day after admission, the patient underwent computed tomographic coronary angiography (CT-CAG) to determine the spatial relationships between the long LAD and the surrounding structures. It was found that the long LAD originated from the proximal RCA, coursed left between the aortic root and the RVOT then entered the mid anterior interventricular groove, where it gave rise to the D2. The long LAD did not run into the ventricular crest or the septum. The D2 was the dominant artery in the anterior wall of the left ventricle (Figure 2).

The patient was discharged without complications on the fifth day after admission.

## Conclusions

Dual LAD anomalies have been classified into six different types based on the origin and course of the long LAD (Table 1) [1,2]. In types I, II and III, both the long and the short LADs originate from the proximal LAD. In types IV, V and VI the long LAD originates from the proximal RCA or from the right coronary sinus. In types IV and V, the long LAD takes either an epicardial course or an intramyocardial course within the septal crest, while in type VI which has recently been described by Maroney and Klein [1], the long LAD courses between the RVOT and the aortic root. Type VI dual LAD anomaly may have greater clinical significance than other types because compression of the coronary artery between the RVOT and the aortic root in situations of increased pulmonary blood flow could cause coronary blood flow restriction and sudden cardiac death [3]. Although bridging phenomenon was not observed in the traversing portion of the long LAD, dynamic obstruction could be more accurately evaluated using intravascular ultrasound [4]. To our knowledge, this is the second reported case of type VI dual LAD anomaly. The course of the long LAD in this case is similar to that in the previous report, but there are two minor morphological differences. First, the RCA ostium, morphologically similar to the LMCA is high take-off, points downward, and is located underneath the RVOT. Second, the long LAD enters the anterior interventricular groove at a more basal septum, allowing it to give rise to the D2.

**Table 1:** **Classification of dual left anterior descending coronary arteries [**[1]**]**

**Type**	**Origin**		Course of long LAD^*^	**Major branches**	
	**Short LAD**	**Long LAD**		**Short LAD**	**Long LAD**
I	Proximal LAD	Proximal LAD	Epicardial course on the LV side of the proximal AIVG, reentering the distal AIVG	Septal	Diagonal
II	Proximal LAD	Proximal LAD	Epicardial course on the RV side of the proximal AIVG, reentering the distal AIVG	Septal and diagonal	
III	Proximal LAD	Proximal LAD	Intramyocardial course in the proximal septum, then either emerging epicardially in distal AIVG or terminating intramyocardially as septal branches.	Diagonal	Septal
IV	LMCA	Proximal RCA	1. Epicardial course anterior to the RVOT continuing to the distal AIVG	Septal and diagonal	
			2. Intramyocardial course within septal crest emerging epicardially in the distal AIVG		
V	LCS	RCS	Intramyocardial course within the septal crest emerging epicardially in the distal AIVG	Septal and diagonal	
VI	LMCA	Proximal RCA	Epicardial course between the RVOT and the aortic root, continuing to the mid or distal AIVG	Septal and diagonal	Diagonal

- The course of the short LAD is along the proximal AIVG in all types.

- *LAD*, left anterior descending artery; proximal LAD, the portion of the LAD from bifurcation of the left main coronary artery to the first diagonal branch; AIVG anterior interventricular groove; *LV*, left ventricle; *RV*, right ventricle; *LMCA*, left main coronary artery; proximal RCA, RCA from the ostium to the first curved portion; *LCS*, left coronary sinus; *RCS*, right coronary sinus; *RVOT*, right ventricular outflow tract.

It is difficult to determine the course of an anomalous coronary artery using angiography alone, especially when the anomalous artery passes between the great vessels or through the myocardium [5]. CT-CAG can be a powerful tool in assessing spatial relationships between an anomalous coronary artery and surrounding structures [6,7]. In this case, CT-CAG was useful in determining the course of the long LAD. The reconstructed images, from which the RVOT was removed, showed that the long LAD passed between the RVOT and the aortic root without taking an intramyocardial course (Figure 2B).

Unlike the previous case, our patient underwent PCI. Although the MP and the JR 4.0 guiding catheters were suitable for coaxial positioning with the downward-pointing RCA and the traversing portion of the long LAD, they did not provide good support. Deep intubation as used in this case is one way to improve the backup force in transradial interventions [8]. Alternatively, a buddy wire technique, buddy balloon technique, different choice of guiding catheter, or transfemoral approach could improve support [9,10].

In summary, type VI dual LAD is a recently recognized rare anomaly in which the long LAD courses between the RVOT and the aortic root, potentially leading to negative clinical consequences. CT-CAG can provide spatial information on the relationship between long LAD and the neighboring structures. Successful PCI can be performed safely in this type of anomaly with accurate assessment of the coronary anatomy and appropriate selection of hardware.

## Consent

Written informed consent was obtained from the patient for publication of this case report and accompanying images. A copy of the written consent is available for review from the Series Editor of *BMC Cardiovascular Disorders*.

## Abbreviations

PCI: Percutaneous coronary intervention; LAD: Left anterior descending artery; RCA: Right coronary artery; RVOT: Right ventricular outflow tract; LMCA: Left main coronary artery; D1: First diagonal artery; D2: Second diagonal artery; CT-CAG: Computed tomographic coronary angiography.

## Competing interests

The authors declare that they have no competing interests.

## Authors’ contributions

YL, the first author, performed diagnostic coronary angiography for the patient, wrote the manuscript and made the illustrations. YHL, the corresponding author, performed percutaneous coronary intervention for the patient and participated in three-dimensional reconstruction of computed tomographic coronary angiogram. JS participated in the design of the report and helped to draft the manuscript reporting the first case of percutaneous coronary intervention in a particular coronary anomaly. KSK participated in the design of the report and the version to submit. All authors read and approved the final manuscript.

## Pre-publication history

The pre-publication history for this paper can be accessed here:

http://www.biomedcentral.com/1471-2261/12/101/prepub
